# Kinetic mechanisms of electron bifurcation with electron transfer flavoprotein, NADH, butyryl-CoA dehydrogenase, and ferredoxin reveal a semiquinone cycle

**DOI:** 10.1016/j.jbc.2025.110727

**Published:** 2025-09-16

**Authors:** Jeerus Sucharitakul, Montisa Mangkalee, Pattarawan Intasian, Soraya Pornsuwan, Ulrich Ermler, Wolfgang Buckel, Pimchai Chaiyen

**Affiliations:** 1 Department of Biochemistry, Bangkok, Thailand; 2 Center of Excellence in Integrative Immuno-Microbial Biochemistry and Bioresponsive Nanomaterials, Faculty of Dentistry, Chulalongkorn University, Bangkok, Thailand; 3 Department of Chemistry, Faculty of Science, Chulalongkorn University, Bangkok, Thailand; 4 School of Biomolecular Science and Engineering, Vidyasirimedhi Institute of Science and Technology (VISTEC), Rayong, Thailand; 5 Department of Chemistry and Center of Excellence for Innovation in Chemistry, Faculty of Science, Mahidol University, Bangkok, Thailand; 6 Department of Molecular Membrane Biology, Max Planck Institute of Biophysics, Frankfurt am Main, Germany; 7 Laboratorium für Mikrobiologie, Fachbereich Biologie and Synmikro, Philipps-Universität, Marburg, Germany; 8 Max-Plank-Institut für terrestrische Mikrobiologie, Marburg, Germany

**Keywords:** flavin-based electron bifurcation, *Acidaminococcus fermentans*, electron transfer flavoprotein (EtfAB), anionic FAD semiquinone, rapid kinetics, ferredoxin, flavodoxin, butyryl-CoA dehydrogenase, crotonyl-CoA

## Abstract

Electron transfer flavoprotein (EtfAB, with α-FAD and β-FAD) and tetrameric butyryl-CoA dehydrogenase (Bcd, with δ-FAD in each subunit) from *Acidaminococcus fermentans* catalyze electron bifurcation which reduces low potential ferredoxin (Fd) and high potential crotonyl-CoA using NADH as an electron donor. Our previous rapid kinetic studies have demonstrated “pseudo-electron bifurcation” where NADH and two EtfAB molecules generate EtfA_SQ_B (A_SQ_ contains α-FAD^•−^) and the charge-transfer complex of EtfA_SQ_B_HQ_:NAD^+^ (B_HQ_ contains β-FADH^−^). Since the radical in EtfA_SQ_B inhibits the further reduction of β-FAD with NADH, the question arises as to how the five components of the complete system interact to mediate the whole flavin-based electron bifurcation. This study shows that Bcd releases the inhibition effect of α-FAD^•−^, allowing fast β-FAD reduction for turnover. In the presence of both Bcd and Fd, the total β-FADH^−^ of EtfAB bifurcates to afford α-FAD^•−^ and Fd^−^; a second bifurcation yields α-FADH^−^ in the Bcd-EtfA_HQ_B complex and additional Fd^−^. In the presence of crotonyl-CoA, two simultaneous one-electron transfers from both EtfA_HQ_B yield reduced Bcd and two EtfA_SQ_B, confirmed by electron paramagnetic resonance spectroscopy. This step is proposed to require a slow conformational change of the Bcd-EtfAB complex for electron transfer with a limiting rate constant of 0.0098 s^−1^ at 4 °C, but increases about 14-fold to 0.14 s^−1^ at 30 °C, the optimal growth temperature of *A. fermentans*. The final reduction of crotonyl-CoA to butyryl-CoA completes the cycle, which we call the semiquinone cycle of electron bifurcation, because it starts and ends with a semiquinone.

An enzyme system from the anaerobic bacterium *Acidaminococcus fermentans* catalyzes the bifurcation of an electron pair derived from NADH (1). One electron reduces the high-potential crotonyl-CoA, which drives the reduction of the low-potential ferredoxin (Fd) or flavodoxin. The enzyme system comprises of an electron transfer flavoprotein (EtfAB) with two subunits and homotetrameric butyryl-CoA dehydrogenase (Bcd). This system and that from *Megasphaera elsdenii* (2, 3) have the advantages of containing no iron-sulfur clusters and an Etf separated from Bcd whereas in the bifurcating systems of Clostridia, Etf is tightly attached to Bcd (4). Thus, *A. fermentans* Etf can be prepared under air, and the interaction of Etf with Bcd can be studied. Three differently bound FAD molecules are involved in this system: α-FAD on EtfA (subunit A), β-FAD on EtfB (subunit B), and δ-FAD on each subunit of the tetrameric Bcd.

To analyze this five-component system, containing EtfAB, Bcd, Fd, NADH, and crotonyl-CoA, we started to study the reduction of EtfAB with NADH, which led to two reduced EtfAB monomers, one containing α-FAD^•−^ and β-FAD (EtfA_SQ_B) and the other Etf monomer containing α-FAD^•−^ with a charge-transfer complex (CTC) of NAD^+^ and β-FADH^−^ (EtfA_SQ_B_HQ_:NAD^+^) (Fig. 1*A*) (5). For this process, we coined the expression “pseudo-electron bifurcation”. This mechanism has been heavily criticized by Russ Hille et al (6). They wrote that a mixture of EtfA_HQ_B_HQ_ with EtfAB resulted in no visible spectral change within 10 min. Hence, Hille concluded that there was no intermolecular electron transfer between EtfA_HQ_B_HQ_ and EtfAB, especially between β-FADH^−^ of EtfA_HQ_B_HQ_ and α-FAD of EtfAB. But in pseudo-electron bifurcation, the intermolecular electron stems from the semiquinone of β-FAD (*E*°′ ∼ −700 mV), which has a much higher energy than one electron of β-FADH^−^ (*E*°′ ∼ −300 mV) (7).

**Figure 1:**
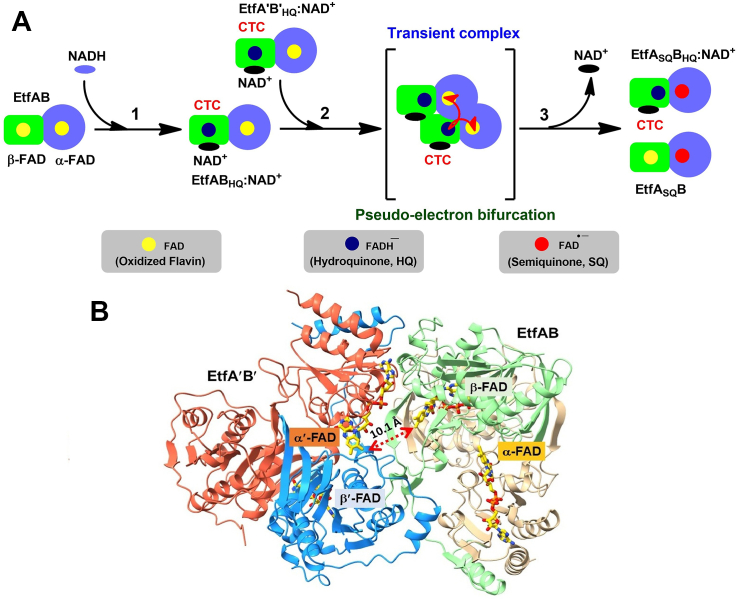
**Pseudo-electron bifurcation in EtfAB_HQ_.** The *yellow*, *red*, and *blue circle* represents oxidized, semiquinone, and reduced form of flavin, respectively. The *red half-head arrow* represents a one-electron transfer. *A,* pseudo-electron bifurcation occurs after the reduction of β-FAD of EtfAB with NADH to EtfAB_HQ_ (step 1) which forms the charge-transfer complex (CTC) β-FADH^−^:NAD^+^. One EtfAB_HQ_ transiently interacts with another molecule (EtfA′B′_HQ_) (step 2). One β-FADH^−^ bifurcates intermolecularly one electron to α′-FAD, and the other electron intramolecularly to α-FAD followed by CTC decay (step 3). *B*, model for the transient EtfAB-EtfA′B′ complex. The picture shows the shortest possible distance between β-FAD and α′-FAD of ca. 10 Å using the Coot program (10) for modeling. EtfA′B′ is colored *orange* (α′-subunit) and *blue* (β′-subunit), and EtfAB is shown in *green* (β-subunit) and *beige* (α-subunit). EtfAB, electron transfer flavoprotein; EtfAB_HQ_, Etf containing β-FADH^-^ and α-FAD.

We further found that α-FAD^•−^ inhibited the reduction of β-FAD with NADH in the same Etf molecule. Due to the radical, the reduction of EtfA_SQ_B with NADH occurred at a 1500-fold lower rate than the reduction of EtfAB (5). The question arises of how an efficient electron bifurcation can work with such slow and inefficient steps.

In this article, we analyze various combinations of the components, which reveal unexpected interactions and demonstrate that an efficient electron bifurcation can only occur in the presence of all five components.

## Results

### Pseudo-electron bifurcation in EtfAB

It was previously shown that mixing EtfAB with a stoichiometric amount NADH resulted in a rapid reduction of β-FAD, yielding the CTC of EtfAB_HQ_:NAD^+^ (step 1, Fig. 1*A*). According to spectroscopic data, the complex decays to EtfA_SQ_B and the CTC of EtfA_SQ_B_HQ_:NAD^+^ states (Steps 2 and 3, Fig. 1*A*) at a rate constant of 44 s^−1^ (5). Although one β-FAD returns to the oxidized state, a second β-FADH^−^ remains reduced in a CTC with NAD^+^. This finding was interpreted as an intramolecular one-electron transfer from β-FADH^−^ to α-FAD and an intermolecular one-electron transfer from β-FADH^−^ to α′-FAD of a second EtfAB_HQ_:NAD^+^ molecule (termed EtfA′B′_HQ_:NAD^+^ with α-FAD′ and β-FAD′). This process was designated as pseudo-electron bifurcation by us because both acceptors have identical reduction potentials (8). The pseudo-electron bifurcation is different from a classic electron bifurcation, which is a high-potential reduction that drives a low-potential reduction, *i.e.*, the reduction of Fd or flavodoxin with NADH (9). Therefore, the transient interaction of two EtfAB molecules is possible in such a way that two EtfAB molecules should be oriented close to each other with the distance between β-FAD and α′-FAD shorter than 14 Å. Using the Coot program (10) to render a minimal intermolecular overlap, a pseudo-electron bifurcation model was built by rotating and translating a second molecule EtfA′B′ relative to the fixed molecule EtfAB, resulting in a postulated transient complex with a β-FAD – α′-FAD distance of ca. 10 Å (Fig. 1*B*). As the distances between β-FAD and intramolecular/intermolecular α-FADs are not accurately determined, it remains open whether the high-potential electron of β-FADH^−^ flows first to α-FAD or α-FAD′.

### Interaction of EtfAB with Bcd

**Figure 2:**
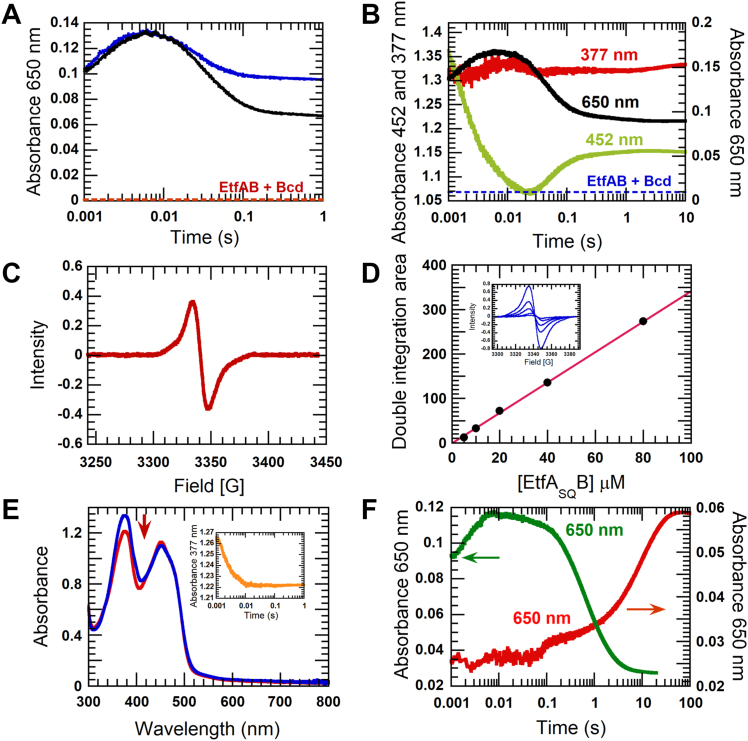
**Interaction of Bcd with EtfAB.***A*, *blue line*: 40 μM EtfAB is mixed with 40 μM NADH. *Black line*: the same reaction as *A*, but in the presence of 20 μM Bcd. Bcd causes a larger CTC decay at 650 nm. The *dotted-red line* represents a baseline absorbance of 40 μM EtfAB + 20 μM Bcd at 650 nm before mixing with NADH. *B,* the same reaction as *A* in the presence of 20 μM Bcd. The reaction is monitored at 377 nm for semiquinone (*red line*), 452 nm for oxidized flavin (*green line*), and 650 nm for CTC (*black line*). The *dotted-blue line* represents a baseline absorbance of *B* at 650 nm before mixing with NADH. *C*, the EPR spectrum of reaction *B*. *D*, the quantitation of α-FAD semiquinone (α-FAD^•−^) using EPR spectra. The different concentrations of EtfAB: 5 μM, 10 μM, 20 μM, 40 μM, and 80 μM, were titrated with sodium dithionite under anaerobic condition using microtitrater attached to tonometer. The enzyme concentration was determined using the extinction coefficient at 452 nm (see Experimental procedures). The reductive titration was monitored using spectrophotometer until the semiquinone peak at 377 nm reached to maximum absorbance to generate EtfA_SQ_B. The EPR signals were obtained from the same running condition as described (see Experimental procedures). Areas under curve of each EPR spectrum (inset *D*) from double integration were plotted against EtfA_SQ_B concentrations. This calibration curve is a reference to estimate α-FAD^•−^ concentrations. *E*, the spectral perturbation of the semiquinone from the interaction with Bcd. The *blue line* is the summation of the absorption spectrum of 40 μM EtfA_SQ_B (prepared by reductive titration using sodium dithionite) and the absorption spectrum of 20 μM Bcd. The *red line* is the spectrum obtained after mixing EtfA_SQ_B with Bcd at the same concentrations as above. The *blue spectrum* shows a decrease in absorbance at 377 nm as compared to the *red spectrum* (as indicated by the *red arrow*). A decreased absorbance at 377 nm from mixing 40 μM EtfA_SQ_B with 20 μM Bcd was monitored using a stopped-flow spectrophotometer (inset *E*). *F*, the inhibition of the reduction of β-FAD by α-FAD^•−^ is released by Bcd. *Red line*: 40 μM EtfA_SQ_B is mixed with 40 μM NADH. The kinetic trace at 650 nm shows the slow formation of the CTC from the inhibition effect of α-FAD^•−^ on the reduction of β-FAD. *Green line*: 40 μM EtfA_SQ_B is equilibrated with 20 μM Bcd for 50 s before mixing with 40 μM NADH. The kinetic trace shows fast formation and decay of the CTC β-FADH^-^:NAD^+^. The arrows indicate the y-axes of the kinetic traces. Bcd, butyryl-CoA dehydrogenase containing δ-FAD; EtfAB, electron transfer flavoprotein; EPR, electron paramagnetic resonance; EtfA_SQ_B, Etf containing α-FAD^•^^-^; EtfA_HQ_B, Etf containing α-FADH^-^ and β-FAD; CTC, charge-transfer complex.

In the presence of Bcd and EtfAB, the CTC decay qualitatively follows the same kinetics as in the absence of Bcd. However, the decay mediated by pseudo-electron bifurcation is larger than without Bcd (blue *versus* black line, Fig. 2*A*), of which 41% CTC decay has been demonstrated (5). Measurement of the absorbencies in the presence of Bcd revealed exactly 50% EtfA_SQ_B and 50% CTC of EtfA_SQ_B_HQ_:NAD^+^ (black line, Fig. 2B). The maximum absorbance at 650 nm of the CTC at 6 ms is 0.160 ± 0.001 (using a dotted-blue line as a baseline), exactly 2.0 times as high as the end of the decay of 0.080 ± 0.005 (black line, Fig. 2*B*). Thus, Bcd stabilizes Etf through the conformational interaction of the EtfA part with the active site of Bcd, 8 Å apart from δ-FAD (11). Though the intensity of the semiquinone at 377 nm does not change during the reaction, as indicated by the red line in Figure 2*B*, the presence of the semiquinone is demonstrated by a strong electron paramagnetic resonance (EPR) signal (Fig. 2*C*). However, EtfAB shows an intrinsic EPR signal with an unusually narrow peak of around 7.3 G (red line, Fig. S1, *A* and *E*), whereas the EPR signal of normal flavin anionic semiquinone has been reported to have a bandwidth of 14 to 15 G (12). The EPR signals at the end of all reactions have characteristics of anionic flavin semiquinone with an average bandwidth of 12.7 G (green line, Fig. S1*E*). Therefore, the intrinsic signals disappear without their contributions at the end of the reactions (Fig. S2).

The quantitation of EPR red semiquinone signal from the calibration curve using different EtfA_SQ_B in range of 5 to 80 μM (Fig. 2*D*) determines concentration of α-FAD^•−^ of the reaction in Figure 2*C* as 39.68 or ∼40 μM which is almost the same as expected value of 40 μM. The result shows strong evidence to confirm that in presence of Bcd, all 40 μM EtfAB_HQ_ molecules a pseudo-electron bifurcation complex to generate 20 μM EtfA_SQ_B (50% CTC decay) and 20 μM EtfA_SQ_B_HQ_:NAD^+^ (CTC), compared to pseudo-electron bifurcation without Bcd (Rx1, Fig. S2) in which the quantitated semiquinone is only 24 μM.

The structure of Bcd in complex with EtfAB from *Clostridioides difficile* shows that four EtfAB molecules interact with a core tetrameric Bcd (11). In the EtfAB-Bcd complex of *A. fermentans*, the molar ratio of EtfAB:Bcd 2:1 is confirmed by mixing 40 μM EtfAB with varied Bcd concentrations plus 40 μM NADH. The effect of Bcd on the larger decay of CTC reaches a maximum of 50% CTC decay at a Bcd concentration of 20 μM (Fig. S3). Therefore, one tetrameric Bcd is proposed to interact with two EtfAB molecules in the EtfAB-Bcd complex of *A. fermentans*.

Another experiment demonstrates a different aspect of the interaction between EtfAB and Bcd. After mixing EtfA_SQ_B with Bcd in a 2:1 ratio, the absorbance of the semiquinone at 377 nm decreases (Fig. 2*E*) within 10 ms by about 5% (inset Fig. 2*E*). Upon binding of EtfAB to Bcd, α-FAD^•−^ moves over a distance of 35 Å from β-FAD (14 Å apart) towards δ-FAD (8 Å apart) inside Bcd (11). The semiquinone is probably somehow shielded in the interior of Bcd. This finding explains why the kinetic trace at 377 nm in Figure 2*B* remains almost constant. A decrease in absorbance caused by the addition of Bcd (Fig. 2*E*) subtracts from an increase in absorbance due to pseudo-electron bifurcation. The quantitative determination of α-FAD^•−^ in Figure 2*C* confirms the complete binding of Bcd to α-FAD^•−^, not Bcd causing disappearance or destabilizing the semiquinone.

The most significant effect of the interaction of EtfAB with Bcd is the abolishment of the 1500-fold inhibition of the reduction of EtfA_SQ_B with NADH (Fig. 2*F*). Apparently, the radical and not the negative charge causes inhibition (5).

### The interaction of EtfAB with Fd

The interaction between EtfAB and Fd causes more CTC decay than pseudo-electron bifurcation (red *versus* black line, Fig. S4*A*). The rate constant of the interaction of EtfAB with Fd using double mixing with varied age times has been determined as ≥ 138 s^−1^ (black lines, Fig. S4*A*), comparable to the observed rate constant of EtfAB reduction with NADH of ∼450 s^−1^. Therefore, Fd and NADH can compete for binding to EtfAB as a kinetic random order type (Steps 1–4, Fig. S4*B*). The kinetic trace at 650 nm shows a CTC decay with the rate constant of 44 s^−1^ (Fig. S4*A*), the same decay is observed with EtfAB and Bcd (Fig. 2*A*). At 5 s, the larger CTC decay at 650 nm in the presence of Fd is 0.0062 (red *versus* black, Fig. S4*A*) indicating that only 7% of EtfAB bifurcates with Fd (complex IV, Fig. S4*B*), while 46% undergoes pseudo-electron bifurcation (complex V, Fig. S4*B*). Therefore, the electron bifurcation between EtfAB and Fd might not be catalytic relevant. Both CTC paths contribute to 53% CTC decay, as shown in Figure S4*B*.

### Interaction of EtfAB + Bcd with Fd

**Figure 3:**
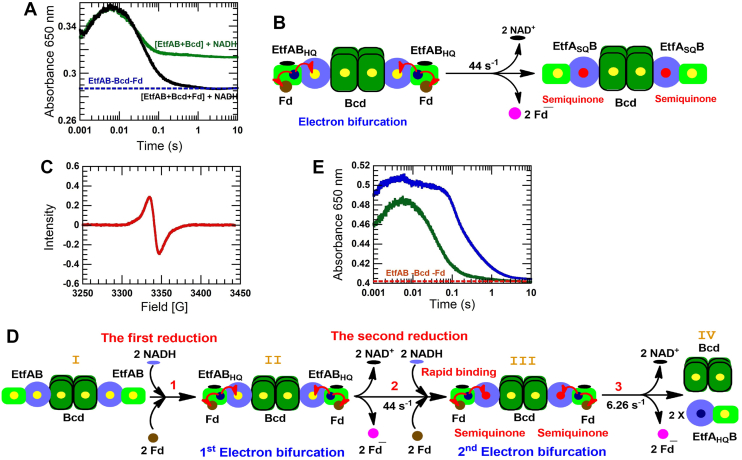
**Electron bifurcation of EtfAB in the presence of both Bcd and Fd.** The green component indicates one tetrameric Bcd (*dark-green*) interacting with two dimeric EtfABs (*light-green and blue*). *A*, a preequilibrated solution of 40 μM EtfAB, 20 μM Bcd, and 40 μM Fd is mixed with 40 μM NADH (*black line*), the same reaction without Fd (*green line*). The *blue-dotted line* represents the baseline absorbance of mixture *A* before mixing with NADH. The results show an almost 100% decay of the CTC in the presence of both Fd and Bcd. *B*, the electron bifurcation of the ternary complex, which results in Fd reduction and EtfA_SQ_B formation, is illustrated. *C*, the EPR spectrum of reaction *A* is used to detect the anionic flavin semiquinone of EtfA_SQ_B. *D,* the first and second electron bifurcation of the ternary complex under two-fold NADH to EtfAB is illustrated. Initially, 40 μM NADH reduce EtfAB (step 1) and cause the first electron bifurcation with Fd reduction. The remaining 40 μM NADH immediately reduce the reoxidized β-FAD, but the reduction rate constant is limited by the rate constant of the preceding electron bifurcation step (step 2). EtfA_SQ_B_HQ,_ generated from the second reduction, bifurcates electrons to reduce the semiquinone and Fd (step 3). *E*, the preequilibrated solution of 40 μM EtfAB, 20 μM Bcd, and 80 μM Fd is mixed with 40 μM NADH (*green line*) or with 80 μM NADH (*blue line*). Both kinetic traces at 650 nm show 100% CTC decay using a baseline at 650 nm of the absorption spectrum of the same mixture as described before mixing with NADH (*red dotted line*). EtfAB, electron transfer flavoprotein; Bcd, butyryl-CoA dehydrogenase containing δ-FAD; Fd, ferredoxin; CTC, charge-transfer complex; EtfA_SQ_B, Etf containing α-FAD^•^^-^; EPR, electron paramagnetic resonance.

Mixing of 40 μM EtfAB, 20 μM Bcd, and 40 μM Fd with 40 μM NADH again forms a CTC with maximum absorbance at 650 nm, 6 ms after reduction. In contrast to previous experiments, the CTC completely decays to the blue dashed baseline after 1 s (Fig. 3*A*, black line). Since both Fd and Bcd have to be present to get a complete CTC decay, a reaction model is likely consistent with bifurcating β-FADH^−^:NAD^+^; one electron goes to Fd generating Fd^−^ and the other to α-FAD generating EtfA_SQ_B with a rate constant of 44 s^−1^ (Fig. 3*B*). The EtfAB-Bcd complex is illustrated as one tetrameric Bcd interacting with two EtfAB molecules (see Fig. S3). The EPR spectrum at the end of the reaction also confirms the semiquinone of EtfA_SQ_B (Fig. 3*C*).

The EPR spectrum in Figure 3*C* yields 39.77 μM semiquinone or ∼40 μM. The model in Figure 3*B* proposes that all 40 μM β-FADH^−^:NAD^+^ molecules bifurcate one electron to Fd and one to α-FAD. Therefore, both 100% CTC decay and quantitated semiquinone are a strong evidence for supporting the model in Figure 3*B*.

Previous considerations suggest that only the hydroquinone rather than the semiquinone can transfer one electron to Bcd (13) because the one-electron reduction potential of α-FAD is much more positive (*E*°′ = +134 mV) than that of α-FAD^•−^ (*E*°′ = −36 mV) (8). Therefore, α-FAD has to be reduced to the hydroquinone, which is achieved in a second electron bifurcation by addition of another 40 μM NADH and 40 μM Fd yielding 40 μM EtfA_SQ_B (step 2, Fig. 3*D*) and 40 μM Fd^−^ (step 3, Fig. 3*D*). The second reduction of EtfA_SQ_B is possible because Bcd releases the inhibition by α-FAD^•−^ (Fig. 2*F*). The reduction is also limited by the first electron bifurcation (step 2, Fig. 3*D*), as shown in the lag phase (6 ms – 0.1 s) before CTC decay (blue line, Fig. 3*E*). However, the rate constant of the CTC decay has significantly decreased from 44 s^−1^ to 6.3 ± 0.1 s^−1^ (green *versus* blue line, Fig. 3*E*). It has been demonstrated that NAD^+^ can inhibit the electron bifurcation (5). Therefore, one reason for the slower rate constant of the second electron bifurcation could be NAD^+^, released from the first electron bifurcation.

### Reactions of crotonyl-CoA with Bcd

**Figure 4:**
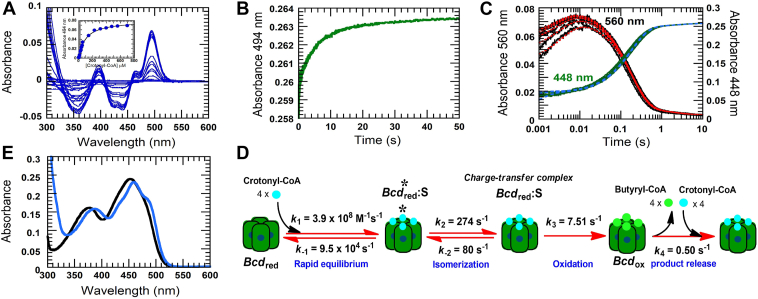
**Binding of crotonyl-CoA to oxidized and reduced Bcd.***A*, to determine *K*_d_ of the Bcd:crotonyl-CoA complex with a double beam spectrophotometer, both reference and sample channels contained 1.5 μM Bcd. The sample cell was titrated with crotonyl-CoA, and an equal-volume buffer was added to the reference cell. The absorbance differences at 494 nm were plotted against the crotonyl-CoA concentrations (inset *A*). *B*, the kinetics of crotonyl-CoA binding to Bcd. A total of 2.5 μM Bcd was mixed with 6 mM crotonyl-CoA (saturation concentration to Bcd). The kinetics were monitored by the absorbance change at 494 nm to obtain an observed rate constant for crotonyl-CoA binding to Bcd. *C*, oxidation of reduced Bcd with crotonyl-CoA. Four micrometers of Bcd reduced with dithionite to the hydroquinone (δ-FADH^‒^) is mixed with increasing crotonyl-CoA concentrations (0.48 mM, 0.96 mM, 1.92 mM, 3.84 mM, and 8 mM). The kinetic traces at 560 nm (*black-solid lines*) show the formation and decay of a CTC intermediate, whereas the kinetic traces at 448 nm (*green-solid lines*) show flavin reoxidation. The lower to upper traces are from low to high crotonyl-CoA concentrations. The *dotted red lines* and *blue lines* are simulation traces according to the model in *D*. *D*, the kinetic mechanism of *Bcd*_red_ oxidation with crotonyl-CoA (S) starts with rapid-equilibrium binding of S to *Bcd*_red_ to form the Michaelis–Menten complex (*Bcd*∗_red_*:S*) followed by isomerization to the CTC *(Bcd*_red_*:S*, absorbing at 560 nm). The oxidation step is the hydride transfer from δ-FADH^‒^ to crotonyl-CoA, yielding butyryl-CoA. The last step is a slow releasing product butyryl-CoA from active site limiting rapid rebinding of excess crotonyl-CoA. *E*, the absorption spectrum of reoxidized Bcd bound crotonyl-CoA shows characteristic of shoulder during 450 to 500 nm at 10 s (*blue line*), compared with free Bcd (*black line*). Bcd, butyryl-CoA dehydrogenase containing δ-FAD; CTC, charge-transfer complex.

The interaction of crotonyl-CoA with oxidized Bcd shows obvious spectral perturbation of Bcd during 300 to 520 nm with the highest absorbance change at 494 nm (Fig. 4*A*). The dissociation constant (*K*_d_) has been determined by static titration of crotonyl-CoA into Bcd. The *K*_d_ of crotonyl-CoA binding to Bcd is calculated from a plot of absorbance change at 494 nm *versus* crotonyl-CoA concentrations as 95 ± 6 μM (inset Fig. 4*A*). The observed rate constant for binding of crotonyl-CoA to Bcd under saturation concentration is determined from rapid mixing of Bcd with 6 mM crotonyl-CoA. The reaction is monitored from the absorbance change at 494 nm (green line, Fig. 4*B*). The observed rate constant is analyzed as 35 ± 0.3 s^−1^, which will be compared with crotonyl-CoA binding to reduced Bcd in the next paragraph.

The oxidation of reduced Bcd with crotonyl-CoA is performed in a stopped-flow apparatus. The reactions show an increase in absorbance at 560 nm within dead time (black lines, Fig. 4*C*). These exponential phases are slightly dependent on crotonyl-CoA concentrations, whereas the kinetic traces at 448 nm for δ-FADH^−^ oxidation still show a reduced state (green lines, Fig. 4*C*). Hence, δ-FADH^−^ and crotonyl-CoA form a CTC with an absorbance maximum at 560 nm, which decay in the second exponential phase (12–1000 ms). This phase is consistent with a large increase in absorbance at 448 nm, the oxidation of δ-FADH¯ with crotonyl-CoA. The CTC formation shows saturation kinetics because the kinetic traces at 560 nm almost superimpose at high concentrations of crotonyl-CoA. The saturation kinetics implies a two-step process for CTC formation: rapid equilibrium binding of reduced Bcd (Bcd_HQ_; Bcd containing δ-FADH^−^) to crotonyl-CoA (S) forms the Michaelis–Menten complex (step 1, Fig. 4*D*) followed by isomerization to the CTC of δ-FADH¯:crotonyl-CoA (step 2, Fig. 4*D*). Then, crotonyl-CoA is reduced to butyryl-CoA (step 3, Fig. 4*D*). Finally, the slow kinetic phase (1–10 s) of 0.50 s^−1^ with slightly increasing absorbance at 448 nm or slightly decreasing absorbance at 560 nm (Fig. 4*C*) is independent of crotonyl-CoA concentrations. This is possibly a slow releasing of butyryl-CoA from Bcd active site whereas excess crotonyl-CoA rapidly binds to reoxidized Bcd because the absorption spectrum of Bcd at 10 s shows the spectral perturbation with shoulder during 450 nm – 500 nm which is characteristic of crotonyl-CoA bound to Bcd (blue line, Fig. 4*E*), compared to the absorption spectrum of free Bcd (black line, Fig. 4*E*). However, this rapid binding is limited by slow butyryl-CoA releasing (step 4, Fig. 4*D*).

Most of the increase in absorbance at 560 nm occurred during dead time. The simulation using the reaction model in Figure 4*D* can estimate each rate constant of the individual steps. The observed *K*_d_ of crotonyl-CoA to the reduced enzyme is calculated as 56 μM from the rate constants obtained from simulation and using Equation 1. This *K*_d_ is not significantly different from that of crotonyl-CoA binding to the oxidized enzyme (95 μM).

(1) $$K_{d}=\frac{\left[{Bcd}_{red}\right]\left[S\right]}{\left[{Bcd}_{red}:S\right]+\left[{Bcd}_{red}:S^{*}\right]}=\frac{k_{-1}/k_{1}}{1+\left(k_{2}/k_{-2}\right)}$$

### Electron transfer from EtfA_HQ_B and EtfA_HQ_B_HQ_ to Bcd with and without crotonyl-CoA

The previous results have elucidated the reaction mechanism for generating fully reduced α-FAD and Fd^−^. In this section, the experiments are designed to evaluate how the electrons are transferred from α-FADH^−^ to δ-FAD of Bcd. The first experiment investigates the difference between the electron transfer from α-FAD semiquinone (α-FAD^•−^) and that from α-FADH^−^ to δ-FAD. Both EtfA_HQ_B and EtfA_SQ_B are mixed with Bcd (Fig. S5). The kinetic trace at 377 nm from mixing EtfA_HQ_B shows semiquinone formation with a rate constant of 0.120 ± 0.002 s^−1^ (blue line, Fig. S5*A*), whereas the kinetic trace at 448 nm for flavin reduction also shows the same kinetics (green line, Fig. S5*A*). No absorbance change of the control reaction from mixing EtfA_HQ_B with anaerobic buffer confirms that the increase in absorbance at 377 nm stems from the presence of Bcd (red line, Fig. S5*A*). In contrast to EtfA_HQ_B, mixing of EtfA_SQ_B with Bcd almost shows no absorbance change at 377 nm (blue line, Fig. S5*B*), 448 nm (green line, Fig. S5*B*), and the control reaction from mixing of EtfA_SQ_B with anaerobic buffer (without Bcd) (red line, Fig. S5*B*). The results demonstrate that only the fully reduced form of α-FAD can transfer one electron to Bcd. Therefore, adding crotonyl-CoA in the following experiment helps to elucidate the mechanism of the electron transfer, because only fully reduced Bcd (δ-FADH^−^) can reduce crotonyl-CoA.

**Figure 5:**
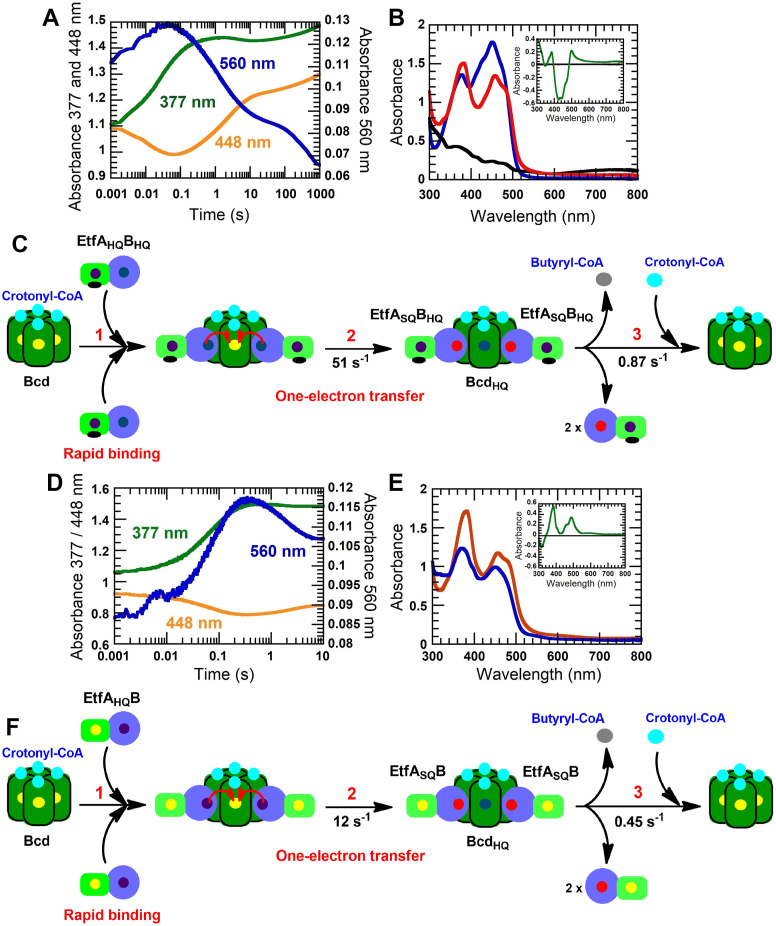
**Electron transfer from α-FADH^−^ to the Bcd:crotonyl-CoA complex.***A*, 40 μM EtfAB (*light-green and blue*) is titrated with NADH until both α- and β-FAD are fully reduced (EtfA_HQ_B_HQ_). The solution EtfA_HQ_B_HQ_ is mixed with 20 μM Bcd (*dark-green*) + 1 mM crotonyl-CoA (*blue circles*). The reaction is monitored at 560 nm for CTC of δ-FADH^−^:crotonyl-CoA complex (*blue line*), 377 nm for semiquinone (*green line*), and 448 nm for flavin oxidation (*orange line*). *B,* the *red spectrum* of reaction *A* taken at 1000 s shows semiquinone formation with a peak at 377 nm. A solution of 40 μM EtfA_HQ_B_HQ_ generated by reduction of EtfAB with NADH yields the *black spectrum*. The *blue spectrum* is the same plus 20 μM Bcd. This spectrum is used as a reference to observe spectral changes at 377 nm for the semiquinone. The difference spectrum (*green line*) between the *red* and *blue lines* shows an increase in absorbance at 377 nm for semiquinone formation (inset *B*). *C,* the mechanism of the electron transfer from EtfA_HQ_B_HQ_ to Bcd is illustrated. The semiquinone at 1000 s indicates a one-electron transfer from two molecules of α-FADH^−^ to form the Bcd_HQ_:crotonyl-CoA complex (step 2) for catalytic reduction of crotonyl-CoA (step 3) to butyryl-CoA (*gray circle*). *D*, the same reaction as (*A*) but in the presence of EtfA_HQ_B (prepared using sodium dithionite). The reaction is monitored at 560 nm for the CTC of the Bcd_HQ_:crotonyl-CoA complex (*blue line*), at 377 nm for semiquinone (*green line*), and at 448 nm for flavin oxidation (*orange line*). *E*, the *blue spectrum* is from mixing 40 μM EtfA_HQ_B with 20 μM Bcd. This spectrum is used as a reference to observe spectral changes at 377 nm for the semiquinone. The *orange* spectrum of reaction *D* was taken at 10 s. The difference spectrum (*green line*) between the *orange* and *blue lines* shows an increase in absorbance at 377 nm for semiquinone formation (inset *E*). *F*, illustration of the mechanism or electron transfer from EtfA_HQ_B to Bcd when β-FAD is in the oxidized state. Bcd, butyryl-CoA dehydrogenase containing δ-FAD; EtfAB, electron transfer flavoprotein; EtfA_HQ_B_HQ_, Etf containing α-FADH^−^ and β-FADH^−^; CTC, charge-transfer complex; EtfA_HQ_B, Etf containing α-FADH- and β-FAD; Bcd_HQ_, Bcd containing δ-FADH^−^.

Mixing of EtfA_HQ_B_HQ_ with Bcd + 1 mM crotonyl-CoA (∼10-fold of *K*_d_ for substrate saturation preforming the complex) shows a fast increase in absorbance at 560 nm (1–40 ms) with a rate constant of 51 ± 1 s^−1^ due to the CTC of δ-FADH^−^:crotonyl-CoA (blue line, Fig. 5*A*). This exponential phase correlates with an increase in absorbance at 377 nm for semiquinone (green line, Fig. 5*A*). The same kinetics at both wavelengths indicate that the semiquinone is formed concurrently with CTC of δ-FADH^−^:crotonyl-CoA. It has been known that the CTC is formed very fast (within dead time, Fig. 4*C*). Therefore, this limiting rate constant of 51 s^−1^ reflects the rate constant of the electron transfer. The change of EtfA_HQ_B_HQ_ (black line, Fig. 5*B*) to the red spectrum during 1000 s indicates that the oxidation of α-FADH^−^ forms the red semiquinone at 377 nm (red line, Fig. 5*B*), as compared to the spectrum of the mixture of EtfAB and Bcd before titration with NADH (blue line, Fig. 5*B*). The peak at 448 nm of the red spectrum is also lower than the blue spectrum, confirming the Bcd reduction.

The results have demonstrated a one-electron transfer from each of the two molecules of α-FADH^−^ to δ-FAD (step 2, Fig. 5*C*), whereas the isomerization step to form the CTC is very fast. Therefore, the CTC is formed with the same limiting rate constant of 51 s^−1^. The semiquinone formation is also correlated with Bcd reduction with a decrease in absorbance at 448 nm (orange line, Fig. 5*A*). In addition, it leads to assume that the electron transfer from EtfA_HQ_B_HQ_ in Figure S5*A* without crotonyl-CoA should have the same mechanism as in the presence of crotonyl-CoA. Its binding to Bcd can accelerate the electron transfer from two molecules of EtfA_HQ_B_HQ_ from 0.120 s^−1^ to 51 s^−1^. The decay of the CTC at 560 nm from 0.04 to 10 s (blue line, Fig. 5*A*) is correlated with an increase in absorbance at 448 nm (orange line, Fig. 5*A*) due to Bcd oxidation by crotonyl-CoA with a rate constant of 0.868 ± 0.005 s^−1^ (step 3, Fig. 5*C*).

To investigate whether the oxidation state of β-FAD has an effect on Bcd oxidation by crotonyl-CoA, EtfA_HQ_B was prepared by reductive titration using sodium dithionite. EtfA_HQ_B was mixed with Bcd + 1 mM crotonyl-CoA. Similar to the previous results, this exponential phase of CTC at 560 nm (blue line, Fig. 5*D*) also correlates with an increase in absorbance at 377 nm for semiquinone (green line, Fig. 5*D*) and also correlates to a decrease in absorbance at 448 nm (orange line, Fig. 5*D*). The kinetic trace at 377 nm increases (1 ms–0.3 s) and then remains stable until 10 s. The spectrum at 10 s also represents the existence of the red semiquinone (orange line, Fig. 5E), compared to the combined spectrum of both EtfA_HQ_B and oxidized Bcd (blue line, Fig. 5E).

The concurrent formation of semiquinone and CTC at 560 nm also confirms a one-electron transfer from each of the two molecules of α-FADH^−^ to δ-FAD (step 2, Fig. 5*F*). The CTC of δ-FADH^−^:crotonyl-CoA is formed with the rate constant of 11.8 ± 0.05 s^−1^ (kinetic trace at 560 nm, blue line, Fig. 5*D*) which is much slower than using EtfA_HQ_B_HQ_. The result shows that the oxidation state of β-FAD has an influence on the rate constant for the electron transfer from α-FADH^−^ to the Bcd:crotonyl-CoA complex. The CTC decay (0.3 s–10 s) at 560 nm (blue line, Fig. 5*D*), which correlates with an increase in absorbance at 448 nm (orange line, Fig. 5*D*), indicates oxidation of Bcd_HQ_ with crotonyl-CoA with a rate constant of 0.453 ± 0.004 s^−1^ (step 3, Fig. 5*F*). The spectrum at 10 s during 400 to 500 nm represents the characteristics of oxidized δ-FAD (red line, Fig. 5*D*). The oxidation of Bcd_HQ_ with crotonyl-CoA without EtfAB (7.6 s^−1^, Fig. 4*D*) is much faster than in the presence of either EtfA_HQ_B_HQ_ or EtfA_HQ_B. Possibly, the interaction of these reduced proteins with the Bcd:crotonyl-CoA complex might affect Bcd oxidation-reduction.

**Figure 6:**
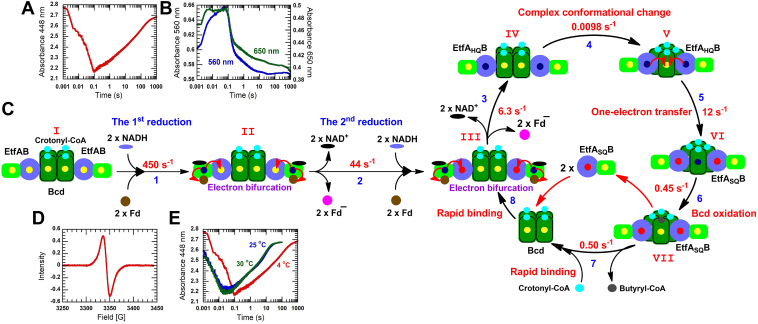
**The catalytic reduction of crotonyl-CoA in the presence of the ternary complex of EtfAB:Bcd:Fd.** The ternary complex of 40 μM EtfAB (*light-green* and *blue*), 20 μM Bcd (*dark-green*), and 80 μM Fd (*brown*) is mixed with 80 μM NADH + 1 mM crotonyl-CoA. *A*, the reaction is monitored at 448 nm (*red line*) for flavin *red* semiquinone (see Fig. S6*C*, Supporting information). *B*, the CTCs of Bcd_HQ_:crotonyl-CoA and EtfAB_HQ_:NAD^+^ is monitored at 560 nm (*blue line*) and at 650 nm (*green line*), respectively. *C*, the catalytic reduction of crotonyl-CoA in the presence of the ternary complex is illustrated. The reaction starts with the first reduction of the EtfAB-Bcd (complex I): Fd and NADH bind to EtfAB-Bcd or EtfAB_HQ_-Bcd in a random order (see Fig. S4*B*, Supporting information). After the first electron bifurcation (complex II), generating (EtfA_SQ_B)_2_-Bcd and 2 Fd^−^, an excess NADH immediately reduces the reoxidized β-FAD to generate complex III (step 2). Two second electron bifurcations generate (EtfA_HQ_B)_2_-Bcd) (complex IV) and 2 Fd^−^ (step 3). The slow conformational change forming complex V (step 4) results in two molecules of EtfA_HQ_B interacting with one δ-FAD of Bcd for simultaneous two one-electron transfers from each EtfA_HQ_B (step 5). The fully reduced Bcd is reoxidized with crotonyl-CoA (step 6). Butyryl-CoA is slowly released from oxidized Bcd active site (step 7). The excess crotonyl-CoA rapidly binds to Bcd with limited rate of butyryl-CoA releasing. Under excess NADH, crotonyl-CoA, and Fd, the resulting EtfA_SQ_B reforms with Bcd:crotonyl-CoA to the ternary complex III (*red arrow* and *black arrows*, step *8*,) and proceeds in the next round of the catalytic cycle. *D*, the EPR spectrum of *A* at 1000 s detects flavin semiquinone. *E*, temperature effect on the conformational change of the protein complex. The same reaction as *A* is performed at different temperatures of 4 °C, 25 °C, and 30 °C (*red*, *blue*, and *green lines*, respectively). The *red line* is replotted the trace at 4 °C in panel A. The rate constant of the one-electron transfer from α-FADH^−^ to δ-FAD is monitored at 448 nm of which the rate constant is limited by the conformational change of complex IV (step 4). EtfAB, electron transfer flavoprotein; Bcd, butyryl-CoA dehydrogenase containing δ-FAD; Fd, ferredoxin; EtfAB_HQ_, Etf containing β-FADH^−^ and α-FAD; EtfA_SQ_B, Etf containing α-FAD^•-^; EtfA_HQ_B, Etf containing α-FADH^−^ and β-FAD; CTC, charge-transfer complex; EPR, electron paramagnetic resonance.

The peak at 377 nm of the spectrum after electron transfer between EtfAB and the Bcd:crotonyl-CoA complex in Figure 5*E* is higher than in Figure 5*B*. Possibly, the increased semiquinone formation indicates a more efficient electron transfer from α-FADH^−^ of EtfA_HQ_B to Bcd. The reduction of crotonyl-CoA also supports that EtfA_HQ_B is more catalytic relevant than EtfA_HQ_B_HQ_ (Fig. 6*C*).

### Catalytic reduction of crotonyl-CoA of the ternary complex of EtfAB:Bcd:Fd

This experiment is to investigate the rate-limiting step when the reaction is initiated by mixing the ternary complex with two-fold NADH to EtfAB. Under this condition, the reaction is expected to proceed in the first electron bifurcation with 40 μM Fd and the second electron bifurcation with the other 40 μM Fd. The first exponential phase of absorbance change at 448 nm shows a fast decrease within dead time (1 ms–6 ms) and continues decreasing until 0.1 s (red line, Fig. 6*A*). The first part of this phase (1 ms–6 ms) is a fast reduction with CTC formation at 650 nm (green line, Fig. 6*B*) (step 1, Fig. 6*C*). Then, the kinetic trace at 650 nm shows a lag phase (6 ms–0.1 s) from the second fast reduction of reoxidized β-FAD, in which the rate constant is limited by the first bifurcation (step 2, Fig. 6*C*). The kinetic trace at 377 nm for semiquinone formation could not be monitored because of the high absorption from using 80 μM Fd. The kinetic trace at 448 nm indicates a surprisingly slow reoxidation with a rate constant of 0.0098 ± 0.0001 s^−1^ (red line, Fig. 6*A*). The rate constant is much lower than the fast β-FAD reduction by NADH (step 1, Fig. 6*C*), the rate constant of the first electron bifurcation with Fd (44 s^−1^) (step 2, Fig. 6*C*), and the second bifurcation with Fd (6.3 s^−1^) (step 3, Fig. 6*C*). In addition, the rate constant for one-electron transfer from each of the two molecules of α-FADH^−^ of EtfA_HQ_B to Bcd:crotonyl-CoA complex is 12 s^−1^ (step 5, Fig. 6*C*). When Bcd_HQ_ is in complex VI (step 6, Fig. 6*C*), the rate constant for crotonyl-CoA reduction is 0.45 s^−1^.

All reaction steps, as described above, have no contribution to this very slow rate constant. Therefore, a slow conformational change of the EtfAB-Bcd complex to complex V (step 4, Fig. 6*C*) could be the most possible reason for the slow oxidation of reduced Bcd. The slow increase of 0.0098 s^−1^ in absorbance at 448 nm should stem from the oxidation of α-FADH^−^ to α-FAD^•−^, not the complete oxidation of reduced flavin. The overlay of the α-FADH^−^ spectrum with that of α-FAD^•−^ reveals an increase in absorbance of both 377 nm and 448 nm (Fig. S5, *A–C*). Although, the kinetic study for binding of crotonyl-CoA to Bcd has demonstrated a random order with preference binding to Bcd_HQ_ (Fig. 4), the oxidation of Bcd_HQ_ with crotonyl-CoA is very slow in multiple turnovers (0.0098 s^−1^). Therefore, Bcd is mostly in the oxidized form during the catalytic cycle, resulting in a complex with crotonyl-CoA before another turnover (step 7, Fig. 6*C*).

The slow decrease in absorbance at both 560 and 650 nm from 0.1 to 100 s (blue and green line, Fig. 6*B*) which has the same kinetics as the slow increase in absorbance at 448 nm (or semiquinone formation) with a rate constant of 0.0098 s^−1^ (red line, Fig. 6*A*) is the oxidation of the Bcd_HQ_:crotonyl-CoA complex generating oxidized Bcd and butyryl-CoA which is the decay of CTC at 560 nm Fig. 4C). In the catalytic cycle, this step (step 6, Fig. 6*C*) is fully limited by the protein complex conformational change (step 4, Fig. 6*C*). Therefore, the slow CTC decay after 0.1 s at both 560 and 650 nm (blue and green line, Fig. 6*B*) has the same kinetics as the slow increase in absorbance at 448 nm with a rate constant of 0.0098 s^−1^ (red line, Fig. 6*A*).

The large magnitude of the peak in the EPR spectrum (red line, Fig. 6*D*) confirms the semiquinone species (EtfA_SQ_B) of complex VI (Fig. 6*C*). The EPR spectrum shows the characteristic of an anionic flavin semiquinone (with a bandwidth of 13.2 G) in the reactions in the presence of crotonyl-CoA (see RxC2, Fig. S2).

To see the semiquinone absorption peak at 377 nm and to observe same kinetics at 448 nm, the high background of Fd is avoided by decreasing the concentrations of the ternary complex by half and remaining the same ratio of 20 μM EtfAB, 10 μM Bcd, and 40 μM Fd. The solution is mixed with 40 μM NADH. The kinetic traces at 377 and 448 nm also show a slow increase in absorbance with the same kinetics (brown and red line, Fig. S7*A*). The spectrum at 100 s shows a semiquinone peak at 377 nm (blue line, Fig. S7*B*), compared to the spectrum of the ternary complex before mixing with NADH (red line, Fig. S7*B*). The difference in absorption of both spectra confirms the semiquinone peak (inset Fig. S7*B*). The rate constant for oxidation of reduced Bcd by crotonyl-CoA is 0.45 s^−1^ (step 6, Fig. 6*C*), which is much faster than the conformational change of the protein complex (0.0098 s^−1^). Therefore, the Bcd_HQ_:crotonyl-CoA complex decays with the same limiting rate constant of 0.0098 s^−1^ (blue line at 560 nm and green line at 650 nm, Fig. S7*A*), similar to Figure 6*B*.

Rapid kinetics show that the rate of catalytic crotonyl-CoA reduction is fully limited by the protein complex conformational change for the interaction of two α-FADH^−^ molecules to one δ-FAD. This rate constant of 0.0098 s^−1^ seems to be not catalytic relevant. However, this slow rate constant has been measured at 4 °C; it increases about 14-fold to the rate constants of 0.105 ± 0.005 s^−1^ at 25 °C and 0.135 ± 0.006 s^−1^ at 30 °C, which approach the optimal growth temperature of *A. fermentans* (14) (Fig. 6*E*).

## Discussion

During electron bifurcation, the semiquinone in Bcd-EtfA_SQ_B recycles, acts as carrier, and forms the semiquinone cycle (Fig. 6*C*). Initial Fd reduction and semiquinone formation is generated by electron bifurcation, where Cr and Bu are abbreviated as crotonyl-CoA and butyryl-CoA, respectively. Four Bcd:Cr in the bracket represent four Bcd subunits in complex with four crotonyl-CoA.

Initial semiquinone formation by electron bifurcation (two EtfA_HQ_B interacting with two Bcd subunits)

$$\text{EtfAB-}\left[\frac{{\left(\text{Bcd}:\text{Cr}\right)}_{2}}{\text{Bcd}:\text{Cr}-\text{Bcd}:\text{Cr}}\right]\text{-EtfAB}+2\text{Fd}+2\text{NADH}\;\to \;{\text{EtfA}}_{\text{SQ}}B-\left[\frac{{\left(\text{Bcd}:\text{Cr}\right)}_{2}}{\text{Bcd}:\text{Cr}-\text{Bcd}:\text{Cr}}\right]-{\text{EtfA}}_{\text{SQ}}B+2{\text{Fd}}^{–}+2{\text{NAD}}^{+}$$

The (Bcd:Cr)_2_ represents two subunits of tetrameric Bcd not interacting with EtfAB.

1. Fd reduction by electron bifurcation (two EtfA_HQ_B interacting with two Bcd subunits)

$${\text{EtfA}}_{\text{SQ}}B-\left[\frac{{\left(\text{Bcd}:\text{Cr}\right)}_{2}}{\text{Bcd}:\text{Cr}-\text{Bcd}:\text{Cr}}\right]-{\text{EtfA}}_{\text{SQ}}B+2\text{Fd}+2\text{NADH}\;\to \;{\text{EtfA}}_{\text{HQ}}B-\left[\frac{{\left(\text{Bcd}:\text{Cr}\right)}_{2}}{\text{Bcd}:\text{Cr}-\text{Bcd}:\text{Cr}}\right]-{\text{EtfA}}_{\text{HQ}}B+2{\text{Fd}}^{–}+2{\text{NAD}}^{+}$$

2. The conformational change of the protein complex (two EtfA_HQ_B interacting with one Bcd subunit): rate-limiting step

$${\text{EtfA}}_{\text{HQ}}B-\left[\frac{{\left(\text{Bcd}:\text{Cr}\right)}_{2}}{\text{Bcd}:\text{Cr-Bcd}:\text{Cr}}\right]-{\text{EtfA}}_{\text{HQ}}B\;\to \;{\text{EtfA}}_{\text{HQ}}B-\left[\frac{{\left(\text{Bcd}:\text{Cr}\right)}_{3}}{\text{Bcd}:\text{Cr}}\right]-{\text{EtfA}}_{\text{HQ}}B$$

The (Bcd:Cr)_3_ represents three subunits of tetrameric Bcd not interacting with EtfAB.

3. Bcd reduction

$${\text{EffA}}_{\text{HQ}}B-\left[\frac{{\left(\text{Bcd}:\text{Cr}\right)}_{3}}{\text{Bcd}:\text{Cr}}\right]-{\text{EtfA}}_{\text{HQ}}B\;\to \;{\text{Etf}A}_{\text{SQ}}B-\left[\frac{{\left(\text{Bcd}:\text{Cr}\right)}_{3}}{{\text{Bcd}}_{\text{HQ}}:\text{Cr}}\right]-{\text{EtfA}}_{\text{SQ}}B$$

4. Crotonyl-CoA reductio

$${\text{EtfA}}_{\text{SQ}}\text{B-}\left[\frac{{\left(\text{Bcd}:\text{Cr}\right)}_{3}}{{\text{Bcd}}_{\text{HQ}}:\text{Cr}}\right]-{\text{EtfA}}_{\text{SQ}}B+Cr\;\to \;2{\text{EtfA}}_{\text{SQ}}B+{\left(\text{Bcd}:\text{Cr}\right)}_{4}+\text{Bu}$$

5. Bcd-EtfA_SQ_B recycling

$$2{\mathrm{EtfA}}_{\mathrm{SQ}}B+{\left(\mathrm{Bcd}:\mathrm{Cr}\right)}_{4}\to {\mathrm{EtfA}}_{\mathrm{SQ}}B-\left[\frac{{\left(\mathrm{Bcd}:\mathrm{Cr}\right)}_{2}}{\mathrm{Bcd}:\mathrm{Cr}-\mathrm{Bcd}:\mathrm{Cr}}\right]-{\mathrm{EtfA}}_{\mathrm{SQ}}B$$

Sum 1 to 5:

$$2\text{NADH}+2\text{Fd}+\text{Crotonyl}-\text{CoA}\;\to \;2{\text{NAD}}^{+}+2{\text{Fd}}^{-}+\text{Butyryl}-\text{CoA}$$

As seen in reactions 1 to 5, all five components such asNADH, EtfAB, Bcd, Fd, and crotonyl-CoA have to be present for a complete electron bifurcation. If one component is absent, no correct outcome is possible. First of all, NADH is the reductant; nothing happens in its absence. Though EtfAB alone can react with NADH, the outcome is an imprecise pseudo-electron bifurcation. With EtfAB, Bcd, and NADH exactly 50% pseudo-electron bifurcation is observed; the remaining 50% form the stable CTC Bcd-EtfAB_HQ_:NAD^+^. Only in the presence of Fd, 100% electron bifurcation happens with EtfA_SQ_B, Bcd, and NADH forming EtfA_HQ_B-Bcd and Fd^−^. Since the hydroquinone EtfA_HQ_B and not the semiquinone of EtfA_SQ_B reduce Bcd with one electron each, two electron bifurcations are necessary, which generate 2Fd^−^ (step 1). The resulting 2EtfA_HQ_B bound to 2Bcd subunits, rearrange in the rate limiting step 2 (*k* ≈ 0.01 s^−1^) with one Bcd subunit to a trimeric -Bcd-EtfA_HQ_B complex, whereby 2 Bcd subunits are liberated. This rearrangement is followed by the reduction of one δ-FAD of Bcd with 2 EtfA_HQ_B to δ-FADH^-^ and affords 2 EtfA_SQ_B (step 3). Crotonyl-CoA oxidizes δ-FADH^-^ yielding butyryl-CoA (step 4). Finally, 2 EtfA_SQ_B, 2 subunits of Bcd and crotonyl-CoA recycle to the starting complex (step 5). Note that the semiquinone EtfA_SQ_B rather than EtfAB is the main reactant in this cycle; therefore, we call it the “semiquinone cycle”.

In our previous work on electron bifurcation with EtfAB, we found that the semiquinone inhibits the reduction of EtfA_SQ_B with NADH. The inhibition is released by interaction with Bcd, as reported in this work. As mentioned above, EtfAB alone fails to work properly in electron bifurcation; it always requires interaction with Bcd. Other examples are the stoichiometric formation and decay of the CTCs between NAD^+^ and β-FADH^−^ as well as between EtfAB and Fd. Hence, EtfAB and Bcd cannot be considered as independent molecules. They always must work together.

As mentioned in the introduction, in *A. fermentans and M. elsdenii,* Bcd and EtfAB are separate molecules, whereas in Clostridia, as are *Clostridium kluyveri* (4)*, Clostridium tetanomorphum* (15) and *C. difficile* (15), Bcd and EtfAB occur as multienzyme complexes. This work demonstrates that tight association of EtfAB and Bcd has no advantage; it just makes the system simpler.

One would assume that the mechanism of electron bifurcation described here should be similar to that proposed by Nguyen *et al.* (16) with the almost identical enzyme system from *M. elsdenii*. Initially, EtfAB-Bcd also has to be reduced to the semiquinone EtfA_SQ_B-Bcd to start the electron bifurcation. However, according to Figure 12 of Nguyen's article, the steps do not occur pairwise but follow one after the other: Reduction of EtfA_SQ_B-Bcd with NADH and electron bifurcation at a rate constant of 2 s^-1^ from β-FADHˉ to α-FAD^•^ˉ yields α-FADHˉ and β-FAD^•^ˉ (17), which reduces Fd. The second reduction with NADH affords β-FADHˉ and one electron reduction of Bcd with α-FADHˉ to the semiquinone δ-FAD^•^ˉ (6, 16). Then the reaction sequence is repeated: second electron bifurcation at a rate constant of 2 s^-1^ to yield α-FADHˉ and β-FAD^•^ˉ, which reduces Fd. Finally, α-FADHˉ reduces δ-FAD^•^ˉ of Bcd to the hydroquinone at a rate constant of 2 s^−1^ (16), which is oxidized with crotonyl-CoA forming butyryl-CoA.

The mechanism described in this work differs in several aspects. First of all, the reductions of the two Fd, as well as the reduction of Bcd, occur in parallel or in pairs. This is different from the EtfAB-Bcd-Fd system from *M. elsdenii* as described above, which requires α-FAD semiquinone for triggering the subsequent electron bifurcation with Fd reduction (6, 16). The electron bifurcation in *A. fermentans* leading to the reduction of Bcd requires α-FADHˉ, which is formed by the reduction of EtfA_SQ_B-Bcd and Fd with NADH. These reactions have to occur twice, forming 2 Bcd-EtfA_HQ_B, which have to reorganize in the slow rate-limiting step to EtfA_HQ_B-Bcd-EtfA_HQ_B and Bcd. Simultaneous two one-electron transfers yield reduced δ-FADHˉ of Bcd, which converts crotonyl-CoA to butyryl-CoA. Notably, the electron bifurcations described in this work are much faster, with rate constants up to 44 s^−1^. Furthermore, the mechanism of Nguyen *et al.* requires a stable semiquinone of δ-FAD in Bcd (17). Though the semiquinone δ-FAD^•^ˉ has been detected, whether its stability lasts as long as two electron transfers (8), each with a rate constant of 2 s^−1^, has not been reported.

## Experimental procedures

### Reagents and chemicals

NADH (purity ≥ 95%), FAD (purity ≥ 95%), FMN (purity ≥ 93%), crotonic acid anhydride, and imidazole were purchased from Tokyo Chemical Industry. Coenzyme A was purchased from Avanti Polar Lipids. Guanidinium hydrochloride (GuHCl) was purchased from Merck (Calbiochem). Chromatographic media (DEAE-Sepharose, G-25 Sephadex, and IMAC-Sepharose) were purchased from GE Healthcare. Concentrations of the following compounds were determined using the known absorption coefficients: NADH, ε_340_ = 6.3 × 10^3^ M^−1^ cm^−1^ (18); FAD, ε_450_ = 11.3 × 10^3^ M^−1^ cm^−1^ at pH 7.0. Fd from *Clostridium pasteurianum* was prepared as previously described (7), and its concentration was determined based on the known absorption coefficient ε_390_ = 30 × 10^3^ M^−1^ cm^−1^ (19). The flavoproteins EtfAB and Bcd concentrations were determined based on the extinction coefficient of bound FAD, ε_451_ = 10.5 × 10^3^ M^−1^ cm^−1^ and ε_448_ = 12.5 × 10^3^ M^−1^ cm^−1^, respectively (8). According to the method, crotonyl-CoA was synthesized from coenzyme A and crotonic acid anhydride (20). The concentration of crotonyl-CoA was determined based on the known absorption coefficient value ε_263_ = 6.7 × 10^3^ M^−1^ cm^−1^ (21, 22). Each experiment was from the same enzyme purification batch.

The quaternary structures of EtfAB and Bcd are heterodimer and homotetramer, respectively (1). The concentrations of EtfAB and Bcd specified are dimeric and tetrameric, whereas the subunit concentration was calculated based on the extinction coefficient of FAD bound per subunit. Therefore, the subunit concentration of EtfAB and Bcd is divided by 2 and 4, respectively, to obtain dimeric and tetrameric concentrations.

### Preparation of EtfAB, Bcd, and Fd

The genes of EtfAB and Bcd from *A. fermentans* in the vector pET-11a and the gene of Fd from *C. pasteurianum* in the vector pET-22b (containing C-terminal six His-tags) were expressed in ECOS *Escherichia coli* Sonic (Yeast Biotech). To obtain a high yield of Fd, the plasmid pET-22b containing the Fd gene was cotransformed with the plasmid containing the *isc* gene cluster (with tetracycline resistance) for the assembly of Fe-S clusters (23). The bacterial cells with both plasmids were selected on LB agar containing ampicillin (50 μg/ml) and tetracycline (10 μg/ml). The growth of microorganisms for protein production and purification has been described previously (8). EtfAB (1 μM) refers to its dimeric concentration with α- and β-FAD, 1 μM each. Bcd is referred to its tetrameric concentration, of which 1 μM tetramer contains 4 μM δ-FAD. Fd is referred to its monomeric concentration.

### Spectroscopic studies

The absorbance spectra of enzymes and compounds were measured by a double-beam UV-visible spectrophotometer (Shimadzu UV-2550) or Hewlett–Packard diode array spectrophotometer (HP8453) from Agilent Technologies.

### Determination of the K_d_ (dissociation constant) of Bcd and crotonyl CoA from static titration

The spectral perturbation of Bcd from binding to its ligand crotonyl-CoA was analyzed with the double beam UV-visible spectrophotometer, in which both reference and sample cell contained Bcd. The baseline of absorption (300–600 nm) was taken before starting the experiment. Different concentrations of crotonyl-CoA solutions were added to the sample cell, and equal volumes of buffer were added to the reference cell. The *K*_d_ value was determined from the hyperbolic curve of absorbance change against crotonyl-CoA concentrations using Marquardt–Levenberg nonlinear fit algorithms included in KaleidaGraph (Synergy Software 4.5).

### Stopped-flow measurements under anaerobic conditions

The experiments were performed using a TgK Scientific Model SF-61DX stopped-flow spectrophotometer in both single-mixing and double-mixing modes. Anaerobic potassium phosphate pH 7.0 was prepared in a closed tonometer by equilibration with nitrogen (high purity > 99.9%) *via* alternation between evacuation and equilibration for forty cycles using a three-way oblique manifold connected with anaerobic trains and kept in the tonometer under positive pressure with nitrogen gas (∼2.8 × 10^4^ Pa). Solid dithionite in the side arm of the tonometer was mixed with 100 mM anaerobic potassium phosphate pH 7.0 to give 0.5 mg dithionite/ml. The flow system of the stopped-flow instrument was washed and equilibrated with this dithionite solution overnight (∼16–18 h). Before the experiments were performed, the dithionite in the flow system was removed by washing three times with anaerobic 50 mM potassium phosphate pH 7.0. All stopped-flow measurements were performed in anaerobic 50 mM potassium phosphate pH 7.0 at 4 °C.

The solutions of the protein complexes used in the stopped-flow measurements were prepared in a tonometer. The solutions were equilibrated with nitrogen gas under positive pressure by alternated equilibrating and evacuating as described above and kept on ice in a refrigerator overnight (16−18 h). The molar ratios were 2:1 for EtfAB:Bcd and 1:1 for EtfAB:Fd (1).

The dead time of the stopped-flow instrument was 1 to 2 ms. The reactions were monitored using a photomultiplier tube for a selected single-wavelength mode and a charged-couple device detector for spectral changes. Each kinetic trace was from the average of five repeated shots, and each experiment used the same enzyme preparation.

### Preparation of EtfA_SQ_B, EtfA_HQ_B, and EtfA_HQ_B_HQ_

EtfAB in 50 mM potassium phosphate pH 7.0 was transferred into a tonometer with two side arms for the attachment of a quartz cuvette and for the connection to an edACE thread (size #7) with a Michael–Miller adapter (from ACE Glass incorporated). A gas-tight syringe with a long needle (Hamilton) and a microtitrator were filled with the anaerobic reducing reagent (dithionite, 5 mg/ml) and tightly connected to the ACE thread. The enzyme solution was evacuated and equilibrated with nitrogen (high purity > 99.9%) for forty cycles using a three-way oblique manifold connected with anaerobic trains. The anaerobic enzyme solution was kept in the tonometer under positive pressure nitrogen gas (∼2.8 × 10^4^ Pa) and cooled on ice. The enzyme solution was titrated with small amounts of dithionite using a microtitrator until the red semiquinone's maximum absorption peak at 377 nm remained stable. For the preparation of EtfA_HQ_B, the same reductive titration was continued until the peak of the red semiquinone disappeared, and the absorbance at 451 nm decreased to 50% of the starting oxidized spectrum. For the preparation of EtfA_HQ_B_HQ_, the same reductive titration using NADH was carried out until the fully reduced spectrum.

### Detection of flavin semiquinone using EPR

The combination of reactions (Fig. S2, supporting information): 1; (40 μM EtfAB + 40 μM NADH), 2; (40 μM EtfAB + 20 μM Bcd + 40 μM NADH), 3; (40 μM EtfAB + 40 μM Fd + 40 μM NADH) and 4; (40 μM EtfAB + 20 μM Bcd + 40 μM Fd + 40 μM NADH), was mixed with 40 μM NADH (stoichiometric to β-FAD). These 1 to 4 reactions were prepared with a total volume of 1 ml in an anaerobic glove box. The mixture solutions were equilibrated in the glove box for 20 min, and then the desired NADH concentration was added to all reactions. After all reactions were mixed with 40 μM NADH, each was transferred to an EPR quartz tube. The solution in the EPR tube was frozen using an ethanol bath with dry ice in the glove box until the EPR signal was measured.

The combination of reactions in presence of crotonyl-CoA: both reactions of RxC1 and RxC2 contained 1 mM crotonyl-CoA: RxC1; (40 μM EtfAB + 20 μM Bcd + 80 μM Fd + 1 mM crotonyl-CoA + 40 μM NADH), RxC2; (40 μM EtfAB + 20 μM Bcd + 80 μM Fd + 1 mM crotonyl-CoA + 80 μM NADH). The reaction was left for 1000 s after adding NADH in an EPR tube, and then the reaction was quenched by freezing in an ethanol bath.

All EPR tubes with solutions were tightly sealed with a silicone cap and tightly wrapped around with parafilm before being transferred outside the glove box. All frozen solutions in EPR tubes were transferred to the cell block of the EPR machine (ELEXSYS) model PERCH–MU48FX–006(M) cooled with liquid nitrogen at 130 K. The magnetic field was scanned from 3243 to 3443 G.

### Kinetic analysis and simulations

Observed rate constants (*k*_obs_) were obtained from the kinetic traces using exponential fits and the software packages Kinetic Studio (Hi-Tech Scientific). The standard errors of estimation for the best exponential fit to analyze the rate constants are not more than 5.5% of the fitted values. The R^2^ (r square) that provides information about the goodness of fit of the experimental kinetic traces is more than 0.9990.

The observed rate constants from reactions of enzymes with substrates under pseudo-first-order conditions ([substrate] >> [enzyme]) were plotted *versus* substrate concentration to analyze the reaction mechanism using Marquardt–Levenberg nonlinear fit algorithms included in KaleidaGraph (Synergy Software 4.5). Reactions between proteins were not performed under pseudo-first-order conditions because of concentration limitations and high absorption background. The reaction mechanism models and rate constants were obtained from kinetic simulation using enzyme kinetic software Kintex Explorer 6.3 (24, 25). The reaction model and the rate constants obtained from simulation were evaluated using Fitspace analysis (Table S1 in Supporting information) and plotting confidence intervals (Fig. S8 in Supporting information).

## Data availability

All the data are in the manuscript.

## Supporting information

This article contains supporting information (12, 24, 25).

## Conflict of interest

The authors declare that they have no conflicts of interest with the contents of this article.
