# Supplementary material for: Kinetic mechanisms of electron bifurcation with electron transfer flavoprotein, NADH, butyryl-CoA dehydrogenase, and ferredoxin reveal a semiquinone cycle
Source: J Biol Chem. 2025 Sep 16;301(10):110727. doi: 10.1016/j.jbc.2025.110727 (PMC12552937; doi:10.1016/j.jbc.2025.110727)
Supplement: Supporting information [file mmc1.docx]

**Kinetic mechanisms of electron bifurcation with electron transfer flavoprotein, NADH, butyryl-CoA dehydrogenase, and ferredoxin reveal a semiquinone cycle.**

Jeerus Sucharitakul^1,2*^, Montisa Mangkalee^2,3^, Pattarawan Intasian^4^, Soraya Pornsuwan^5^, Ulrich Ermler^6^, Wolfgang Buckel^7,8*^ and Pimchai Chaiyen^4^

^1^Department of Biochemistry and ^2^Center of Excellence in Integrative Immuno-Microbial Biochemistry and Bioresponsive Nanomaterials, Faculty of Dentistry, Chulalongkorn University, Henri Dunant Road, Patumwan, Bangkok 10330, Thailand

^3^Department of Chemistry, Faculty of Science, Chulalongkorn University, Phayathai Road, Patumwan, Bangkok 10330, Thailand

^4^School of Biomolecular Science and Engineering, Vidyasirimedhi Institute of Science and Technology (VISTEC), Wangchan Valley, Rayong 21210, Thailand

^5^Department of Chemistry and Center of Excellence for innovation in Chemistry, Faculty of Science, Mahidol University, Bangkok 10400, Thailand

^6^Department of Molecular Membrane Biology, Max Planck Institute of Biophysics, Max-von-Laue-Str. 3, 60438 Frankfurt am Main, Germany

^7^Laboratorium für Mikrobiologie, Fachbereich Biologie and Synmikro, Philipps-Universität, Marburg, Germany

^8^Max-Plank-Institut für terrestrische Mikrobiologie, Marburg, Germany

**Figure S1. The intrinsic EPR spectra of EtfAB and anionic flavin semiquinone.** The EPR spectra of the flavin semiquinone are measured in 50 mM potassium phosphate, pH 7.0, at a temperature of 130 K. The magnetic field was scanned from 3,243 – 3,443 G (see Experimental procedures). (**A**) The intrinsic EPR signal of 40 μM EtfAB. (**B**) The solutions of 40 μM Fd (blue line) and 20 μM Bcd (green line) give no signal. (**C**) The EPR spectrum of 40 μM EtfAB, 20 μM Bcd, and 40 µM NADH. (**D**) The EPR spectrum of 40 μM EtfAB, 20 μM Fd, and 40 µM NADH (**E**). The bandwidth of the intrinsic EPR signal of EtfAB (red line) is narrower than that of the anionic flavin semiquinone (FAD^•−^) generated by pseudo-electron bifurcation (green line). (**F**) The EPR spectrum from mixing 40 uM EtfAB with excess 5 mM sodium dithionite.

The solution of EtfAB shows an intrinsic EPR signal, which is found in oxidized enzymes without exposure to any reactant (Figure S1A). Bcd and Fd do not contribute to the EPR signal (Figure S1B). The EPR spectrum of the mixture of EtfAB and Bcd (Figure S1C) shows almost the same magnitude as that of EtfAB alone (Figure 1A). Fd causes a slightly higher magnitude (Figure S1D) than EtfAB or a mixture of EtfAB and Bcd. The EPR signal of normal flavin anionic semiquinone has a peak-to-peak bandwidth of 14 – 15 G, whereas some dehydrogenase enzymes show a narrower bandwidth of 12 G for their anionic semiquinone (1). However, the intrinsic EPR signals of S1A, S1C, and S1D show a narrower peak-to-peak bandwidth of an average of 7.3 G (red line, Figure S1E) compared to the EPR spectrum obtained from mixing of 40 μM EtfAB with 40 μM NADH, which generates an anionic semiquinone by pseudo-electron bifurcation (green line, Figure S1E). This EPR spectrum shows in average, a peak-to-peak bandwidth of 12.7 G, similar to the range of the flavin radical dehydrogenases (1).

The EPR spectra of EtfAB taken at the end of the reactions in different combinations with Bcd, Fd, NADH and crotonyl-CoA show all peak-to-peak bandwidths around 12 G (Figure S2), which is broader than the intrinsic EPR signal of 7.3 G. In addition, the magnitude of the EPR spectrum of Rx3 (Figure S2) is lower than the magnitude of the intrinsic EPR signal (Figure S1A). Some reactions, such as Rx2, Rx4, RxC1, and RxC2 (Figure S2), show a higher magnitude of their EPR spectra than the intrinsic EPR signal. These results indicate that the intrinsic EPR signal disappears during the reaction. At the end of the reaction, only the EPR of the red flavin semiquinone is shown.

**Figure S2. The peak-to-peak bandwidth of EPR spectra obtained at the end of reactions with EtfAB.** The EPR spectra at the end of reactions containing EtfAB, Bcd, and Fd (Rx 1 – 4 ) and the reaction with crotonyl-CoA (RxC1 and RxC2). All reactions were performed at a temperature of 130 K. The magnetic field was scanned from 3,243 – 3,443 G (see Experimental procedures).

**Rx1**: 40 μM EtfAB + 40 μM NADH

**Rx2**: 40 μM EtfAB + 20 μM Bcd + 40 μM NADH

**Rx3**: 40 μM EtfAB + 40 μM Fd + 40 μM NADH

**Rx4:** 40 μM EtfAB + 20 μM Bcd + 40 μM Fd + 40 μM NADH

**RxC1**: 40 μM EtfAB + 20 μM Bcd + 80 μM Fd + 1 mM crotonyl-CoA + 40 μM NADH

**RxC2**: 40 μM EtfAB + 20 μM Bcd + 80 μM Fd + 1 mM crotonyl-CoA + 80 μM NADH

**Figure S3. The determination of the molar ratio of EtfAB and Bcd.** 40 μM EtfAB is mixed with varied Bcd concentrations plus 40 μM NADH. The reactions are monitored at 650 nm for CTC decay. Red line: mixing of EtfAB with NADH (without Bcd). The same EtfAB solution is mixed with NADH plus varied Bcd concentrations of 5 μM (blue line), 10 μM (green line), and 20 μM (black line).


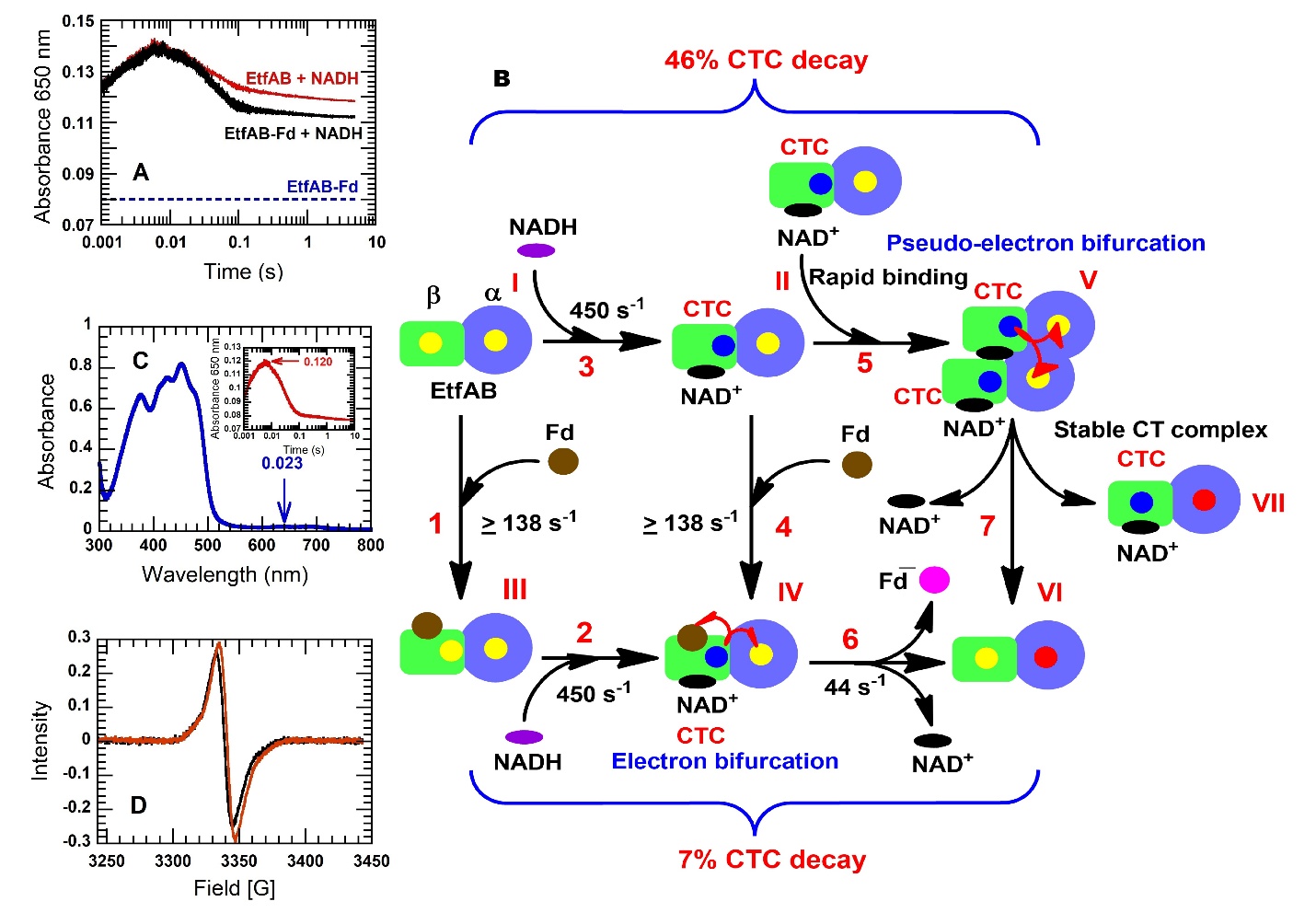


**Figure S4. Interaction of EtfAB with ferredoxin (Fd).** (**A**) Binding of ferredoxin to EtfAB. Black lines: using the double-mixing mode of the stopped-flow machine, 40 μM EtfAB and 40 μM Fd are equilibrated for 0.01 s, 0.02 s, 0.05 s, 0.1 s, 0.2 s, 0.5 s, 1 s and 5 s until addition of 40 μM NADH while the red line (control reaction) is from equilibration of 40 μM EtfAB with anaerobic buffer without Fd for 0.01 s before mixing with 40 μM NADH. The reactions are monitored at 650 nm for CTC formation and decay. The superimposed black lines (thick black line) indicate that EtfAB and ferredoxin are already equilibrated at 0.01 s. (**B**) The illustration shows the electron transfer from EtfAB to Fd. Fd and NADH compete for EtfAB through a random-order mechanism (Steps 1–4). After reduction, most of complex II forms the pseudo-electron bifurcation complex (V), resulting in 46% CTC decay (Step 7), whereas the remaining part of complex II proceeds to electron bifurcation with Fd, resulting in 7% CTC decay (Step 6). (**C**) The figure represents the magnitude of the CTC at 650 nm (from pseudo-electron bifurcation), of which the maximum absorbance (inset) is subtracted by the baseline at 650 nm of 40 M EtfAB before mixing with 40 M NADH (as indicated by the arrow at the blue line spectrum). (**D**) Red line: EPR spectrum from mixing the ternary complex of 40 μM EtfAB + 40 μM Fd with 40 μM NADH. Black line: EPR spectrum from mixing 40 μM EtfAB with 40 μM NADH to generate pseudo-electron bifurcation.

To investigate the binding of EtfAB to Fd, the double mixing mode of the stopped-flow spectrophotometer is used. Solutions of EtfAB and ferredoxin are equilibrated for eight different time intervals between 10 ms and 1 s before mixing with NADH. Absorbance at 650 nm is monitored for CTC formation and decay (Figure S4A). All traces from the different equilibration times can be superimposed well as a single thick black line, indicating that ferredoxin binding to EtfAB is already complete at 10 ms. The observed rate constant for the association of EtfAB and Fd is calculated from the lowest incubation time of 10 ms as ≥ 138 s^−1^, comparable to the observed rate constant of the reduction with NADH of ~450 s^−1^. Therefore, ferredoxin and NADH can compete for binding to EtfAB as a kinetic random order type (Steps 1 – 4, Figure S4B). For comparison, 40 μM EtfAB is mixed with 40 μM NADH in the absence of ferredoxin (red line, Figure S4B). The absorbance difference between the presence and absence of ferredoxin during 0.1 and 1 s indicates that ferredoxin enhances the CTC decay, similar to Bcd.

In the presence of 40 µM ferredoxin, an additional ΔA_650_ = 0.0062 ± 0.0002 of CTC decays (difference of absorbance at 5 s between red and black line, Figure S4A). When a solution of 40 µM EtfAB is mixed with anaerobic buffer, the absorption spectrum shows A_650_ = 0.023 as baseline (blue line as indicated by the arrow, Figure S4C). The same solution, EtfAB, is mixed with 40 µM NADH. The absorption reaches its maximum A_650_ = 0.120 at 6 ms (red line, inset Figure S4C). The total ΔA_650_ (from baseline of 0.023, blue line, Figure S4C) is 0.097 ± 0.002, corresponding to 40 μM CTC.  The molar CTC decay of EtfAB involving Fd can be calculated from 0.0062 / 0.097 × 40 μM = ~2.6 μM. This calculation indicates that only ~2.6 μM Fd proceeds by electron bifurcation (Step 6, Figure S4B), whereas ~37 μM EtfAB proceeds by pseudo-electron bifurcation (Steps 3, 5, and 7, Figure S4B). Thus, pseudo-electron bifurcation contributes to the CTC decay with ½ × 37 μM (18.5 μM) or 46%. The electron bifurcation with Fd contributes 2.6 μM or 7% to the total CTC decay. Therefore, the total CTC decay is 21.1 µM = 18.5 μM + 2.6 μM or 53% is observed.

The EPR spectrum at the end of the reaction shows flavin semiquinone (red line, Figure S4D). The magnitude of the peak is only slightly higher than that of pseudo-electron bifurcation (black line, Figure S4D), because only the additional 2.6 μM semiquinone stems from real bifurcation with Fd.

**Figure S5. The redox states of α-FAD in the electron transfer to Bcd.** 40 μM EtfA_HQ_B and EtfA_SQ_B were prepared by stoichiometric titration of EtfAB with sodium dithionite. (**A**) 40 μM EtfA_HQ_B is mixed with 20 μM Bcd. The reaction is monitored at 377 nm for semiquinone formation (blue line) and at 448 nm for flavin reduction (green line). The red line is a control reaction of mixing 40 μM EtfA_HQ_B with anaerobic buffer. The arrows point to the y-axis belonging to the kinetic trace.  An increase in absorbance at 377 nm indicates a one-electron transfer from α-FADH^−^ to Bcd. (**B**) 40 μM EtfA_SQ_B is mixed with 20 μM Bcd. The reaction is monitored at the same wavelengths as **A**.  The red line is a control reaction of mixing 40 μM EtfA_SQ_B with anaerobic buffer. There is no absorbance change at 377 nm during the same time range as in **A**.

**Figure S6. The spectra of α-FADH^−^ and α-FAD^•−^.** (**A**) Red line: 40 μM oxidized EtfaB (Etf containing only β-FAD). Blue line: the reduced spectrum of EfaB using sodium dithionite as a reductant. (**B**) Red line: 40 μM oxidized EtfAB. Green line: EtfAB is reduced with sodium dithionite to the maximum semiquinone form at 377 nm for obtaining the EtfA_SQ_B spectrum. Blue line: the fully reduced spectrum EtfA_HQ_B_HQ_. (**C**) Black line: The spectrum of 40 μM EtfA_HQ_B_HQ_ (containing 40 μM α-FADH^−^ and 40 μM β-FADH^−^) is subtracted from that of 40 μM EtfaB_HQ_ to obtain the spectrum of EtfA_HQ_. Orange line: The spectrum of 40 μM EtfA_SQ_B is subtracted from that of 40 μM EtfaB to obtain the spectrum of EtfA_SQ_. The overlay of both spectra shows an increase in the absorbencies at 377 nm and 448 nm due to the change from α-FADH^−^ to its semiquinone. Therefore, the red semiquinone is also monitored at 448 nm.

**Figure S7. The catalytic reaction of the ternary complex in the presence of 1 mM crotonyl-CoA.** To avoid the high absorbance at 377 nm, the concentrations of EtfAB, Bcd, and Fd are decreased. (**A**) 20 μM EtfAB, 10 μM Bcd, and 40 μM Fd are mixed with 40 μM NADH. The reaction is monitored by measuring the absorbance changes at 377 nm for the red semiquinone (brown line), at 448 nm for the oxidized flavin (red line), at 560 nm for the CTC of Bcd_HQ_:Crotonyl-CoA (blue line), and at 650 nm for the CTC of FADH^−^:NAD^+^ (green line). (**B**) The spectrum of the reaction in **A** taken at 100 s shows semiquinone formation of EtfA_SQ_B after Bcd reduction (see Step 5 in the catalytic cycle, Figure 6C) with an increase in absorbance at 377 nm (blue line), compared to the spectrum of all components before mixing with NADH (red line). (Inset **B**) The difference spectrum between the blue and red lines confirms an increase in absorbance at 377 nm (green line).

**Reliability of the simulation model for the oxidation of Bcd by crotonyl-CoA.**

***Fitspace analysis***

The reaction model and the rate constants obtained from the simulation were evaluated using Fitspace analysis. The computed Fitspace 1D shows the best fit of the rate constants obtained from the simulation model (2, 3). The inverse plot of the value Chi^2^/minimum Chi^2^ versus parameter boundaries, with a boundary of 1, yields the best overall fit, as shown in Table S1. The confidence analysis was performed using a simulation program to estimate the rate constants in the reaction model of the oxidation of reduced Bcd using crotonyl-CoA. A four-step model was applied for the re-oxidation of reduced Bcd under pseudo-first-order concentrations of Bcd.


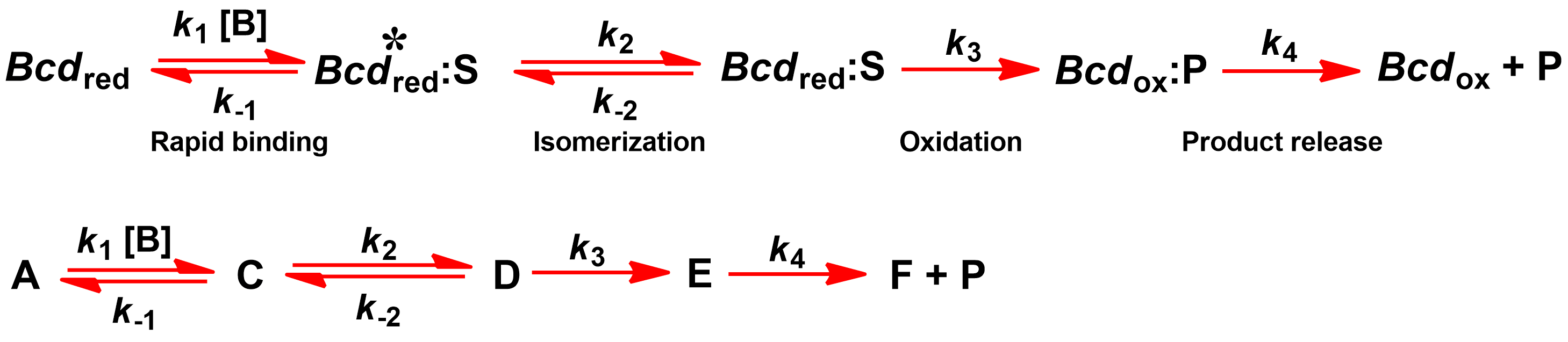


**A** = ${Bcd}_{red}$, **B** = crotonyl-CoA (under pseudo-first-order condition), **C** = ${Bcd}_{red}^{*}:S$, **D** = ${Bcd}_{red}:S$, **E** = ${Bcd}_{ox}:P$, **P** = butyryl-CoA, **F** = ${Bcd}_{ox}$

A, B, C, ......, F are the concentrations at a given time.

The simulation of the absorbance changes at 560 nm for the charge-transfer complex Bcd_red_:S and at 448 nm for Bcd_ox_ and the Bcd_ox_:P complex is shown as Equation S1.

$A=a*A+b*B+c*C+d*D+e*E+f*F+bkg$ Equation S1

Where *a*, *b*, *c*, ......, *f* are extinction coefficients (ε) of A, B, C, ....., F at either 560 nm or 448 nm, and bkg = background or non-zero baseline.

The kinetic traces of the absorbance changes at both 560 and 448 nm from simulation using the model as described above are presented in Figure 4E. The rate constants from the best fit of simulation and the inverse plot of value Chi^2^/minimum Chi^2^ are shown in Table S1.

**Table S1.**

| **Parameter-boundaries at minChi^2^/Chi^2*^ threshold: 0.9804** | | | | |
| --- | --- | --- | --- | --- |
| **Parameters** | **best-fit** | **lower** | **upper** | **Maximum value of minChi^2^/Chi^2^** |
| *k*_1_ (M^−1^s^−1^) | 3.86 × 10^8^ | 1.58 × 10^8^ | 1.23 × 10^10^ | 1.0008 |
| *k*_−1_ (s^−1^) | 9.26 × 10^4^ | 2.78 × 10^4^ | 2.97 × 10^8^ | 1.0007 |
| *k*_2_ (s^−1^) | 274 | 252 | 307 | 1 |
| *k*_−2_ (s^−1^) | 79.5 | 63.6 | 99.4 | 1 |
| *k*_3_ (s^−1^) | 7.51 | 7.13 | 8.03 | 1 |
| *k*_4_ (s^−1^) | 0.499 | 0.131 | 0.976 | 1 |

*min = minimum, Chi^2^ = chi square

***Plotting confidence intervals***

The plot of confidence intervals was performed using KinteK explorer (2, 3).


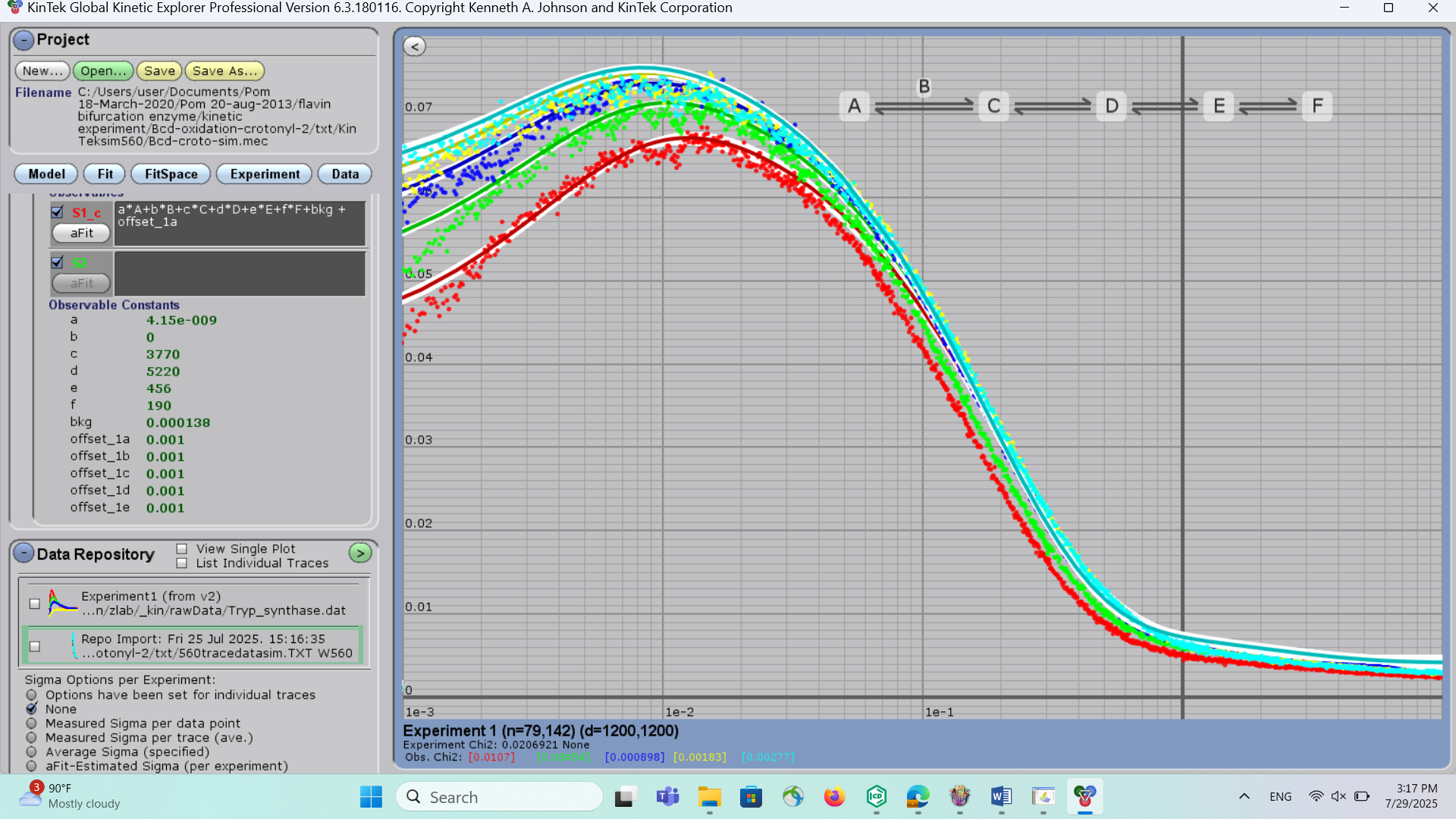


**Figure S8.** The kinetic traces are from the experiment in Figure 4A, with varied crotonyl-CoA concentrations, as shown by the scattered lines. 0.48 mM (red), 0.96 mM (green), 1.92 mM (intense blue), 3.84 mM (yellow), and 8 mM (shade blue). The thick solid lines, colored as described, represent simulation lines based on the model in Figure 4D. The thick white lines are parameter boundaries. The figure illustrates the scattered points of the experimental traces, which are spread around the lines of parameter boundaries, except for the scattered points near the starting collecting time (1–2 ms), as these points are near the dead time.

**References**

1. Edmondson, D. E., Ackrell, B. A., and Kearney, E. B. (1981) Identification of neutral and anionic 8 alpha-substituted flavin semiquinones in flavoproteins by electron spin resonance spectroscopy. *Arch. Biochem. Biophys.* **208**: 69-74

2. Johnson, K. A. (2009) Fitting enzyme kinetic data with KinTek Global Kinetic Explorer. *Methods Enzymol.* **467**, 601-626

3. Johnson, K. A., Simpson, Z. B., and Blom, T. (2009) Global kinetic explorer: a new computer program for dynamic simulation and fitting of kinetic data. *Anal. Biochem.* **387**, 20-29
